# Transcriptome analysis of the fungal pathogen *Rosellinia necatrix* during infection of a susceptible avocado rootstock identifies potential mechanisms of pathogenesis

**DOI:** 10.1186/s12864-019-6387-5

**Published:** 2019-12-26

**Authors:** A. Zumaquero, S. Kanematsu, H. Nakayashiki, A. Matas, E. Martínez-Ferri, A. Barceló-Muñóz, F. Pliego-Alfaro, C. López-Herrera, F. M. Cazorla, C. Pliego

**Affiliations:** 1 Department of Genomics and Biotechnology, IFAPA, Fruticultura Subtropical y Mediterránea, Unidad Asociada de I + D + i al CSIC, Cortijo de la Cruz s/n, 29140 Málaga, Spain; 2 Division of Apple Research, Institute of Fruit Tree and Tea Science, National Agriculture and Food Research Organization (NARO), 92–24 Nabeyashiki, Shimokuriyagawa, Morioka, Iwate, 020–0123 Japan; 3 Present Address: NIFTS, NARO, 2–1 Fujimoto, Tsukuba, 360–8605 Japan; 4 Department of Botany and Plant Physiology, Instituto de Hortofruticultura Subtropical y Mediterránea “La Mayora” (IHSM-UMA-CSIC), Unidad Asociada IHSM-IFAPA, University of Málaga, 29071 Málaga, Spain; 5 Department of Crop Ecophysiology, IFAPA, Fruticultura Subtropical y Mediterránea, Unidad Asociada de I + D + i al CSIC, Cortijo de la Cruz s/n, 29140 Málaga, Spain; 6 Instituto de Agricultura Sostenible, CSIC, Apdo. 4084, 144080 Córdoba, Spain; 7 Department of Microbiology, Instituto de Hortofruticultura Subtropical y Mediterránea “La Mayora” (IHSM-UMA-CSIC), University of Málaga, 29071 Málaga, Spain

**Keywords:** Ascomycete, Effectors, *Persea americana*, Virulence, White root rot

## Abstract

**Background:**

White root rot disease caused by *Rosellinia necatrix* is one of the most important threats affecting avocado productivity in tropical and subtropical climates. Control of this disease is complex and nowadays, lies in the use of physical and chemical methods, although none have proven to be fully effective. Detailed understanding of the molecular mechanisms underlying white root rot disease has the potential of aiding future developments in disease resistance and management. In this regard, this study used RNA-Seq technology to compare the transcriptomic profiles of *R. necatrix* during infection of susceptible avocado ‘Dusa’ roots with that obtained from the fungus cultured in rich medium.

**Results:**

The transcriptomes from three biological replicates of *R. necatrix* colonizing avocado roots (RGA) and *R. necatrix* growing on potato dextrose agar media (RGPDA) were analyzed using Illumina sequencing. A total of 12,104 transcripts were obtained, among which 1937 were differentially expressed genes (DEG), 137 exclusively expressed in RGA and 160 in RGPDA. During the root infection process, genes involved in the production of fungal toxins, detoxification and transport of toxic compounds, hormone biosynthesis, gene silencing and plant cell wall degradation were overexpressed. Interestingly, 24 out of the 137 contigs expressed only during *R. necatrix* growth on avocado roots, were predicted as candidate effector proteins (CEP) with a probability above 60%. The PHI (Pathogen Host Interaction) database revealed that three of the *R. necatrix* CEP showed homology with previously annotated effectors, already proven experimentally via pathogen-host interaction.

**Conclusions:**

The analysis of the full-length transcriptome of *R. necatrix* during the infection process is suggesting that the success of this fungus to infect roots of diverse crops might be attributed to the production of different compounds which, singly or in combination, interfere with defense or signaling mechanisms shared among distinct plant families. The transcriptome analysis of *R. necatrix* during the infection process provides useful information and facilitates further research to a more in -depth understanding of the biology and virulence of this emergent pathogen. In turn, this will make possible to evolve novel strategies for white root rot management in avocado.

## Background

*Rosellinia necatrix* is a soilborne ascomycete, belonging to the order Xylariales, which causes white root rot (WRR) disease in a wide range of commercially important crops and ornamental plants. It has been reported that *R. necatrix* can infect over 170 plant species from 63 genera and 30 families [1], listed in 344 *R. necatrix*-host combinations by the United States Department of Agriculture [2]. This pathogen has a worldwide distribution being able to survive in temperate, tropical and subtropical climates [3–6].

In the Mediterranean region of Spain, WRR is especially damaging due to the co-occurrence of favorable environmental conditions for the development of the fungus and susceptible hosts such as avocado (*Persea americana* Mill.) and mango (*Mangifera indica* L.) [7, 8]. Nowadays it is considered as one of the most important threats affecting avocado productivity [7].

Affected avocado trees show rotten roots and are characterized by a yellowing of the leaves that eventually wilt and ultimately, results in death of the tree. *R. necatrix* root invasion usually occurs by the formation of mycelial aggregates over the root surface which penetrate the root tissues among epidermal and cortical cells and finally, collapse the vascular system of the plant [9]. Neither chemical nor physical methods have proven to be fully effective to control this disease due to the capacity of the fungus to survive in acidic soils as well as to colonize numerous hosts; in addition, the pathogen is quite resistant to drought [4, 7]. Nowadays, the obtainment of tolerant rootstocks appears as the most promising approach to control this disease and efforts are underway to reach this goal [10].To add future developments in disease resistance, systematic analysis of pathogenic fungi’s genomes and transcriptomes has become a top priority. Thus, in recent years, many researchers have addressed transcriptomics studies of plant pathogenic fungi/host interactions [11–13]. The analyses of gene expression profiles associated with the fungal infection provides key sources for understanding fungal biology, leading to the identification of potential pathogenicity determinants [11, 14–17]. Recently, Shimizu et al. [13] provided a 44-Mb draft genome sequence of *R. necatrix* virulent strain W97, in which 12,444 protein encoding genes were predicted. The transcriptome analysis of the hypovirulent strain W97, infected with the megabirnavirus 1 (RNmbv1), revealed that primary and secondary metabolism, as well as genes encoding transcriptional regulators, plant cell wall-degradating enzymes (CWDE), and toxin production such as cytochalasin E, were greatly disturbed in the hypovirulent strain. In another study, the transcriptome analysis of the virulent *R. necatrix* strain (KACC40445) identified 10,616 full-length transcripts among which, pathogen related effectors and CWDE encoding genes were predicted [12]. Data presented in both transcriptomics studies are a valuable resource of genetic information; however, to get a deep insight into pathogenesis of *R. necatrix* a comprehensive transcriptomic analysis of a virulent *R. necatrix* strain interacting with its host is necessary. With this aim, this research addresses the comparison of the transcriptomic profiles of *R. necatrix* during infection of susceptible avocado `Dusa´ roots (RGA) and in vitro growth on PDA (Potato Dextrose Agar) media (RGPDA) using RNA-Seq technology. Functional classification based on assignments to publicly available datasets was conducted, and potential pathogenicity genes related to *R. necatrix* virulence were identified providing a better understanding of the WRR disease.

## Results

### Comparative transcriptome analysis of *R. necatrix* growing on avocado roots vs PDA medium

**Fig. 1:**
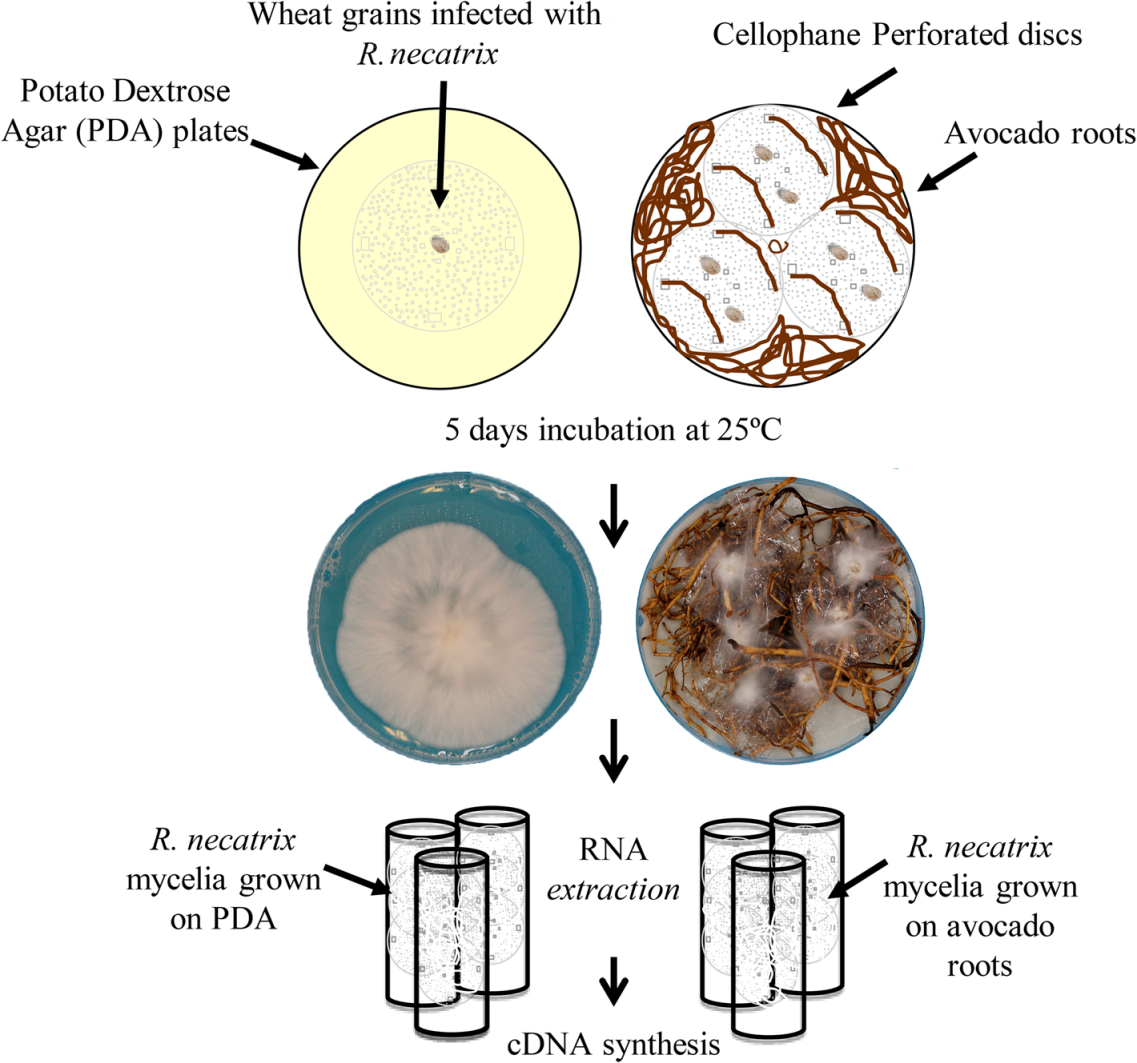
RNA-Seq Experimental Design. Schematic representation of the transcriptome analysis carried out in *R. necatrix* growing on avocado roots in comparison with its growth on Potato Dextrose Agar (PDA) media

**Fig. 2:**
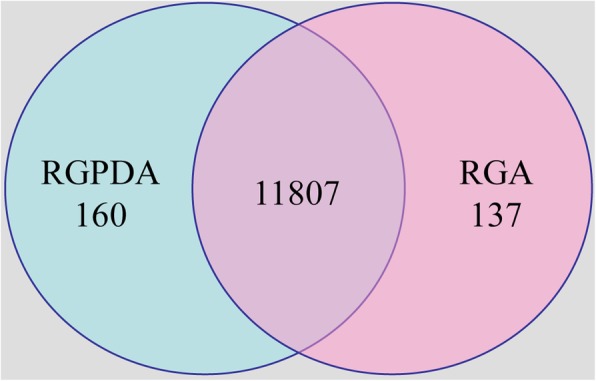
Venn diagram of transcripts expressed during *R. necatrix* growth on avocado roots vs rich medium. Numbers of common and specific transcripts obtained in the transcriptome analysis of *R. necatrix* growing on avocado roots (RGA) in comparison with its growth on Potato Dextrose Agar media (RGPDA). Unique transcripts are shown in only one of the two circles while shared transcripts are illustrated where the circles meet

**Fig. 3:**
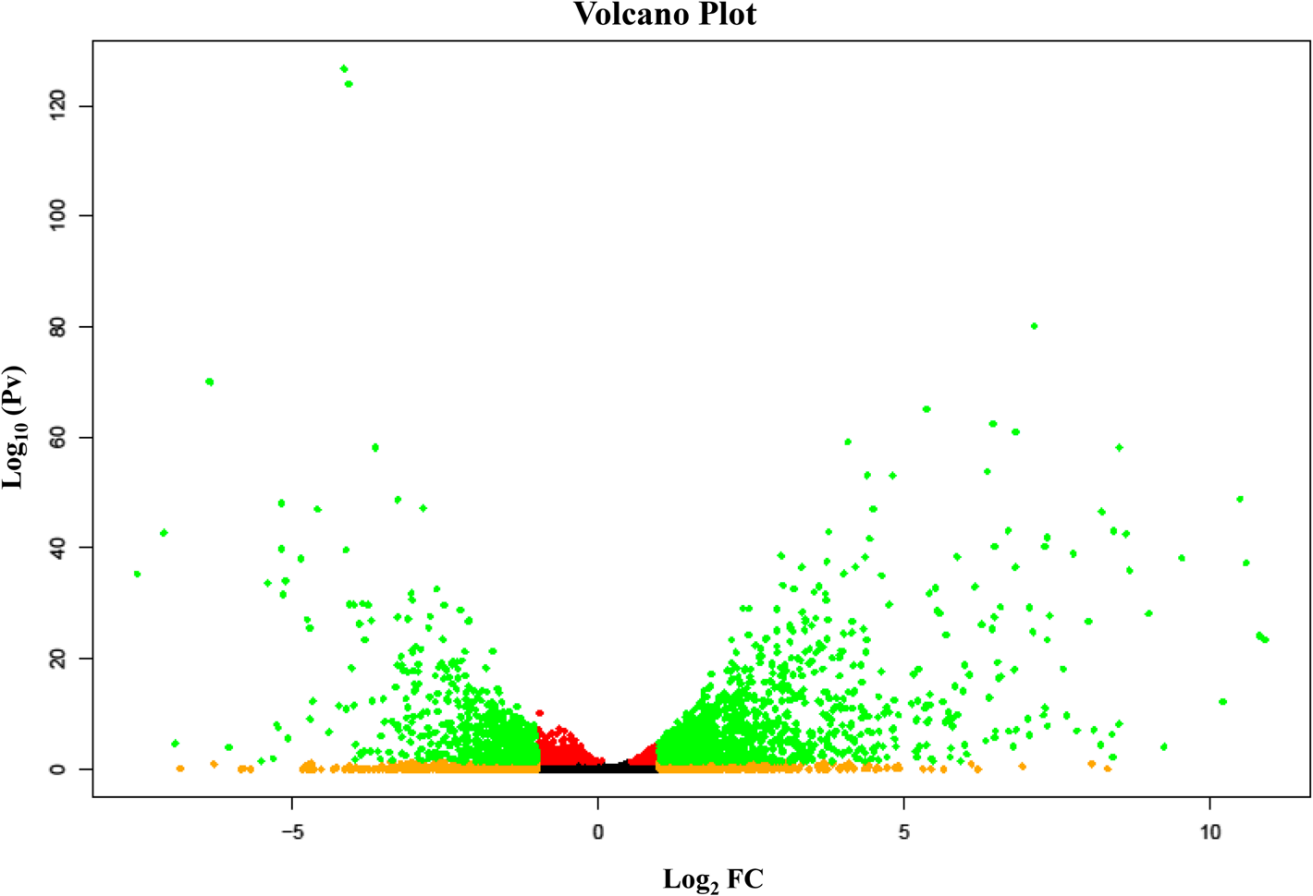
Volcano Plot analysis of differentially expressed genes. Volcano plot summarizing the RNA-Seq DEGs. Significantly up-regulated (right side) or down-regulated (left side) DEGs in *R. necatrix* that also passed the 2 fold-change threshold is shown in green, or in red if the threshold criteria were not met. Non-significantly expressed genes are shown in orange if above or below the fold-change threshold, or black if no criteria were passed

**Fig. 4:**
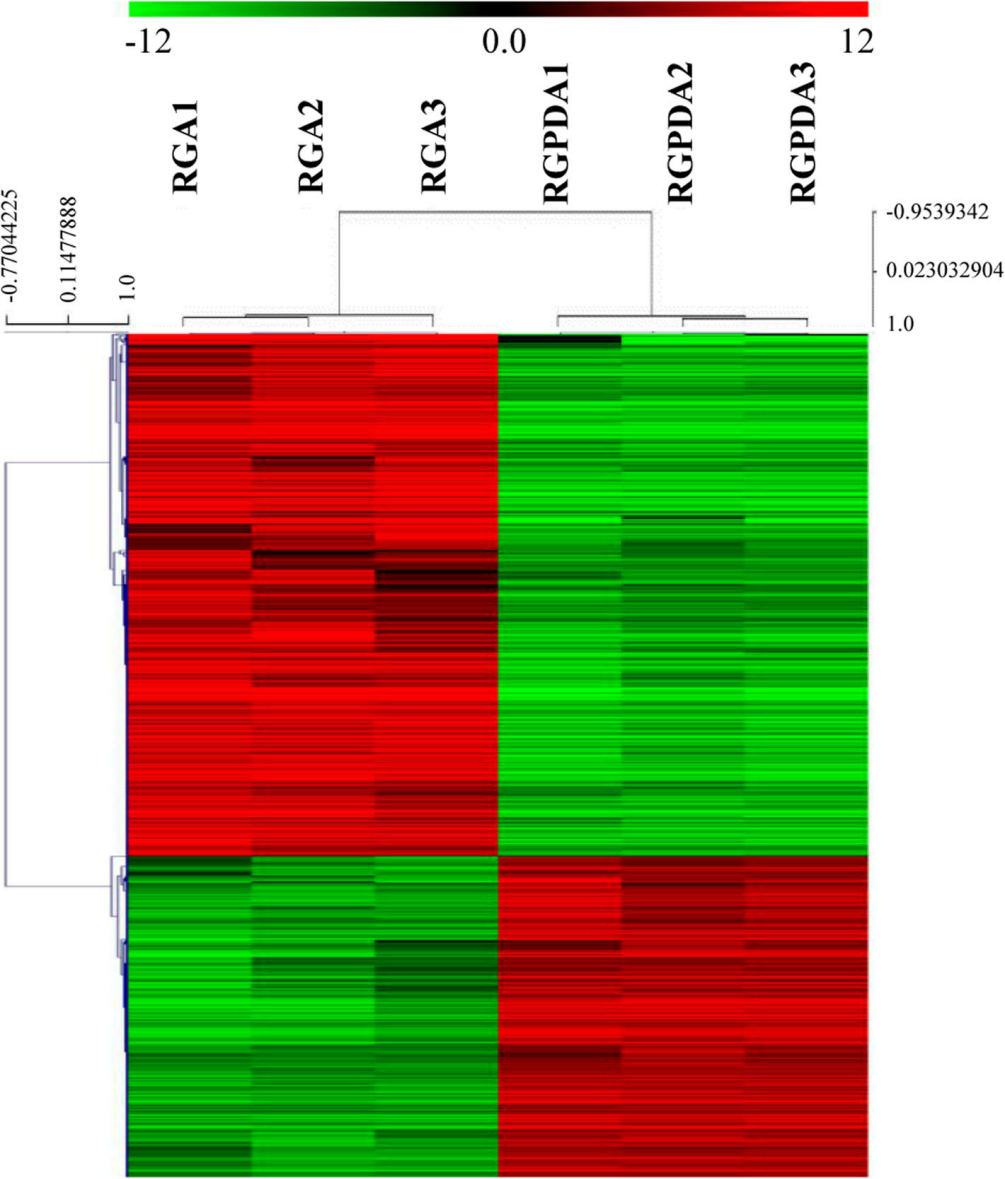
Hierarchical clustering of differentially expressed genes (DEGs). Hierarchical clustering during *R. necatrix* infection on avocado roots (RGA1, RGA2 and RGA3) in comparison with its in vitro growth on Potato Dextrose Agar media (RGPDA1, RGPDA2, RGPDA3). Red and green indicate up- and down regulation, respectively

A transcriptome analysis was carried out to capture genes expressed during *R. necatrix* growth on susceptible `Dusa´ avocado roots and on PDA medium, in order to compare their expression profiles (Fig. 1). The RNA-Seq data including the raw reads from three biological replicates of *R. necatrix* CH53 virulent strain colonizing avocado roots (RGA1; RGA2 and RGA3) and growing on culture medium (RGPDA1; RGPDA2 and RGPDA3) were processed. A total of 12,104 transcripts were obtained, among which 11,807 were present in both conditions, while 137 and 160 transcripts were exclusively expressed in either RGA or RGPDA, respectively (Fig. 2). Total transcripts were subjected to statistical analysis to evaluate differential gene expression between RGA vs RGPDA test situations. Analyses resulted in 1937 differentially expressed genes (DEG), 61.9% induced and 38.1% repressed (− 2 > fold change (FC) > 2; *P*-value < 0.05) (Fig. 3). A heat map of DEGs showed consistence in expression patterns among RGA1, RGA2 and RGA3 and among RGPDA1, RGPDA2 and RGPDA3, supporting the reliability of the RNA-Seq data (Fig. 4).

### Validation of the RNA-Seq analysis

Differences found in gene expression profiles between RGA vs RGPDA were further verified through a quantitative real time PCR (qRT-PCR) assay on total cDNA samples from mycelia of three biological replicates. For this, five randomly selected genes over-expressed in RGA vs RGPDA and with different FC, were analyzed. *Actin* gene was used as reference gene for data normalization. The expression levels of these genes amplified by qRT-PCR are shown in Table 1. Although higher expression values were obtained by qRT-PCR than those observed on the RNA-Seq, results corroborated the overall differences found between the two samples (RGA and RGPDA) in the RNA-Seq analysis.

**Table 1:** qRT-PCR and RNA-Seq expression data of selected contigs over-expressed during *R. necatrix* growth on avocado roots

Gene ID	Description	RGA vs RGPDA	
		qRT-PCR FC^a^	RNA-Seq FC
SAMD00023353_12800020	Related to pisatin demethylase	**838.68**	**90.24**
SAMD00023353_2901300	FAD-binding domain-containing protein	**529.58**	**77.04**
SAMD00023353_2901290	Related to protoporphyrinogen oxidase	**160.78**	**104.04**
SAMD00023353_10000100	Cytochrome p450	**129.64**	**46.61**
SAMD00023353_0800710	Fungal cellulose binding domain	**50.59**	**35.61**

^a^Data are displayed as fold change (FC), calculated by comparing *R. necatrix* growth on avocado roots (RGA) with *R. necatrix* growth on Potato Dextrose Agar medium (RGPDA). The expression data are the mean of three biological replicates. Bold numbers indicate statistically significant results (*t*-Test, *P* < 0.05)

### Functional annotation and pathways analysis of differentially expressed genes (DEGs)

**Fig. 5:**
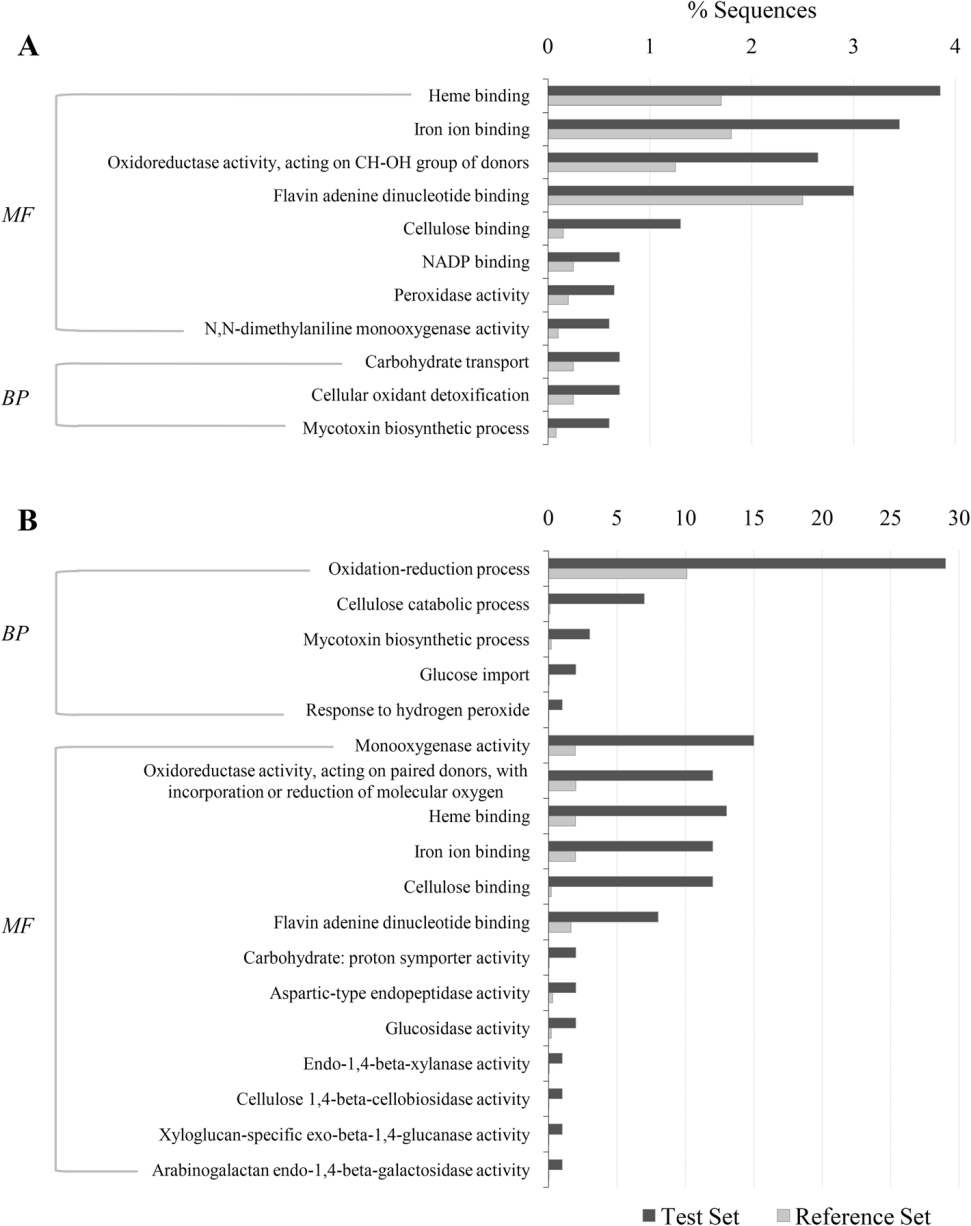
Gene Ontology (GO) enrichment analysis of differentially expressed genes (DEGs). **a**. GO enrichment analysis of DEGs obtained in the transcriptome analysis of *R. necatrix* growing on avocado roots (RGA) in comparison with its growth on Potato Dextrose Agar media (RGPDA). **b**. GO enrichment analysis of the TOP100 DEGs obtained in the transcriptome analysis of RGA vs RGPDA. Enrichment GO terms were obtained by Blast2GO using a cut-off of *P* < 0.05. (BP) biological process; (MF) molecular function

To better understand the infection process of *R. necatrix* colonizing susceptible avocado roots, all differentially expressed genes were functionally enriched and categorized based on blast sequence homologies and gene ontology (GO) annotations using Blast2GO software [18] (*P* < 0.05), selecting the NCBI blast Fungi as taxonomy filter and default parameters. DEGs were significantly grouped into the regulation of eight molecular function (MF), such as heme binding (GO:0020037), iron ion binding (GO:0005506), oxidoreductase activity acting on CH-OH group of donors (GO:0016614), flavin adenine dinucleotide binding (GO:0050660), cellulose binding (GO:0030248), NADP binding (GO:0050661), peroxidase activity (GO:0004601) and N,N-dimethylaniline monooxygenase activity (GO:0004499), and three biological process (BP), such as carbohydrate transport (GO:0008643), cellular oxidant detoxification (GO:0098869) and mycotoxin biosynthesis (GO:0043386) (Fig. 5a). To identify processes and functions over-represented in *R. necatrix* during infection, GO term enrichment analysis was also applied to the Top 100 over-expressed genes (Fig. 5b). The functions of these DEGs were significantly enriched in the regulation of five BP, such as oxido-reduction process (GO:0055114), cellulose catabolic process (GO:0030245), mycotoxin biosynthesis (GO:0043386), glucose import (GO:0046323) and response to hydrogen peroxide (GO:0042542), and 13 MF (Fig. 5b) among which activities related to plant cell wall degradation, including glucosidase activity (GO:0015926); endo-1,4-β-xylanase activity (G0:0031176); cellulose 1,4-beta-cellobiosidase activity (GO:0016162); xyloglucan-specific exo-β-1,4-glucanase activity (GO:0033950) and arabinogalactan endo-1,4-β-galactosidase activity (GO:0031218) were found.To investigate the metabolic pathways affected in *R. necatrix* during avocado root infection, a KEGG pathway analysis was performed with Blast2go [18]. For the total of 1937 DEGs, 100 metabolic pathways that involved 208 genes were identified (*P*-value < 0.05). The metabolic pathways were reorganized into eleven categories (Table 2) being the nucleotides metabolism the one with the highest number of genes (n = 64). Interestingly, metabolic pathways involved in antibiotic and drug metabolism were also affected, in accordance with GO enrichment analysis results, where mycotoxin biosynthetic process was one of the molecular functions over-represented.

**Table 2:** The KEGG pathway analysis using differentially expressed genes (DEGs)

Category	Sequence number^a^
Nucleotides metabolism	64
Organic compounds metabolism	60
Metabolism of cofactors and vitamins	58
Amino acid metabolism	48
Carbohydrate metabolism	42
Antibiotics metabolism	39
Others	37
Drug metabolism	28
Lipid metabolism	24
Energy metabolism	10
Biosynthesis of other secondary metabolites	8

^a^The total number of contigs in each category

### Candidate genes involved in the pathogenesis of *R. necatrix*

At least 69 transcripts showing homology to genes previously reported to be involved in fungal infection were identified among the 1937 DEGs. These include homologs to genes involved in the production of CWDE (Table 3), proteases, fungal toxins, detoxification and transport of toxic compounds, gibberellin biosynthesis and gene silencing (Table 4) as well as gene effectors (Table 5). Out of the 69 selected genes, 30 were associated with cell wall hydrolysis, among which 16 showed fold change (FC) values above 50, with three of them (SAMD00023353_0503130, SAMD00023353_6500680 and SAMD00023353_4001240) allocated in the top20 over-expressed genes in *R. necatrix* during avocado root-colonization (Table 3 and Additional file 1). Five genes were identified as proteases, two aspartic proteases and three serine proteases, with the contig SAMD00023353_1500930 expressed over 411 times in RGA vs RGPDA (Table 4). Five contigs showed homology to genes encoding fungal toxins, among which the contig SAMD00023353_5500610 encoding the putative aflatoxin B1 aldehyde reductase member 2 showed the higher transcript abundance with a FC value of 18.65 (Table 4).

**Table 3:** *R. necatrix* genes encoding cell wall degrading enzymes

Gene ID	Description	Fold Change^a^
Cell Wall Degrading Enzymes		
SAMD00023353_0503130	Putative Glycoside hydrolase family 61 protein	511.82
SAMD00023353_6500680	Glycoside hydrolase family 61 protein	259.37
SAMD00023353_4001240	Glycosyl hydrolase family 43 protein	226.29
SAMD00023353_4000040	Glycoside hydrolase family 128 protein	201.63
SAMD00023353_5900080	Putative glycoside hydrolase family 61	193.76
SAMD00023353_2700270	Putative endoglucanase	166.44
SAMD00023353_10700130	Glycoside hydrolase family 128 protein	162.43
SAMD00023353_3200340	Glycoside hydrolase family 61 protein	155.24
SAMD00023353_0105480	Glycosyl hydrolase family 7	132.66
SAMD00023353_11500050	Cellobiohydrolase II	95.38
SAMD00023353_3900390	Probable endoglucanase	88.85
SAMD00023353_1201160	Glycoside hydrolase family 3 protein	71.42
SAMD00023353_4000610	Glycoside hydrolase family 74 protein	64.97
SAMD00023353_5100270	Glycoside hydrolase family 5 protein	56.75
SAMD00023353_3601090	Putative glycoside hydrolase family 31	54.51
SAMD00023353_1700720	Glycosyl hydrolase family 10	52.82
SAMD00023353_0202710	Glycoside hydrolase family 53 protein	42.82
SAMD00023353_3700510	Glycoside hydrolase family 61 protein	37.07
SAMD00023353_5100760	Glycosylhydrolase family 61–5	36.69
SAMD00023353_0502040	Glycoside hydrolase family 5 protein	28.51
SAMD00023353_1901740	Glycosyl hydrolase family 1	24.70
SAMD00023353_3000290	Glycosyl hydrolase family 16	14.86
SAMD00023353_0204000	Glycosyl hydrolase family 26	11.21
SAMD00023353_7600160	Glycosyl hydrolase family 61	9.35
SAMD00023353_4000450	Glycosyl hydrolase family 18	7.44
SAMD00023353_1601380	Cutinase	7.15
SAMD00023353_0400070	Glycosyl hydrolase family 76	2.77
SAMD00023353_0201450	Glycosyl hydrolase	2.76
SAMD00023353_1002100	Glycosyl hydrolase	2.28
SAMD00023353_0101450	Glycosyl hydrolase	2.24

^a^RNA-Seq fold change calculated by comparing *R. necatrix* growth on roots vs Potato Dextrose Agar

**Table 4:** Genes of *R. necatrix* potentially involved in pathogenesis

Gene ID	Description	Fold Change^a^
Genes Related to Proteases		
SAMD00023353_1500930	Putative acid proteinase protein	411.34
SAMD00023353_6300370	Putative subtilisin-like protein	13.44
SAMD00023353_3200760	Putative subtilisin-like protein	12.19
SAMD00023353_4000440	Putative aspartyl protease	4.82
SAMD00023353_0403740	Related to subtilisin DY	2.60
Genes Related to Toxins Production		
SAMD00023353_5500610	Putative aflatoxin B1 aldehyde reductase member 2	18.65
SAMD00023353_3901210	Putative averantin oxidoreductase	13.89
SAMD00023353_10000060	Putative toxin biosynthesis	4.81
SAMD00023353_1501590	Putative toxin biosynthesis protein	4.30
SAMD00023353_11700090	Sterigmatocystin 8-O-methyltransferase	3.29
Genes Related to Detoxification of Toxic Compounds		
SAMD00023353_5200870	*catA*, catalase	157.78
SAMD00023353_3600430	Putative cycloheximide resistance protein	92.12
SAMD00023353_12800020	Related to pisatin demethylase cytochrome P450	90.24
SAMD00023353_7000300	GMC oxidoreductase	41.39
SAMD00023353_8000300	Beta-lactamase family protein	40.99
SAMD00023353_1100640	GMC oxidoreductase	28.74
SAMD00023353_10400180	Glucose-methanol-choline (GMC) oxidoreductase	21.40
SAMD00023353_10400170	Glucose-methanol-choline (GMC) oxidoreductase	19.54
SAMD00023353_0701730	Putative multidrug resistance protein fnx1	12.69
SAMD00023353_6600160	Beta-lactamase family protein	11.24
SAMD00023353_0702510	Putative ciclohexymide resistance protein	7.18
SAMD00023353_0902760	Putative MFS aflatoxin efflux pump detoxificación	3.87
SAMD00023353_4900180	Putative arrestin domain containing protein	3.40
SAMD00023353_2900030	GMC oxidoreductase	2.77
SAMD00023353_0100280	Putative tetracycline resistance protein TCRI	2.76
SAMD00023353_11800200	Putative drug resistance protein	2.68
SAMD00023353_3200110	Putative pisatin demethylase	2.39
SAMD00023353_3500410	GMC oxidoreductase	2.19
SAMD00023353_2201610	Metallo-beta-lactamase superfamily protein	2.07
Genes Related to Transport of Toxic Compounds		
SAMD00023353_2601150	ABC transporter	7.37
SAMD00023353_2501030	ABC transporter	6.64
SAMD00023353_3000620	ABC-2 type transporter	5.38
SAMD00023353_10000080	Putative MFS multidrug transporter protein	3.28
SAMD00023353_2200710	MFS transporter	2.80
SAMD00023353_6200040	ABC transporter cdr4	2.39
SAMD00023353_7300370	Drug proton antiporter yhk8	2.21
Genes Related to Gibberelin Biosynthesis		
SAMD00023353_10100030	GA4 desaturase family protein	38.02
SAMD00023353_1901120	Gibberellin 20-oxidase	2.39
Gene Related to Gene Silencing		
SAMD00023353_0801000	Argonaute siRNA chaperone complex subunit Arb1	2.38

^a^RNA-Seq fold change calculated by comparing *R. necatrix* growth on roots vs Potato Dextrose Agar

**Table 5:** List of candidate effectors genes in *R. necatrix*

Gene ID	Description	CDS Length	Signal peptide	Effector prediction^a^	Localization
SAMD00023353_2100110	SSCRP protein	923	Yes	0.936	Apoplastic
SAMD00023353_1002580	Hypothetical protein	183	No	0.910	Non-apoplastic
SAMD00023353_3000810	Hypothetical protein	594	Yes	0.890	Apoplastic
SAMD00023353_1201650	Hypothetical protein	400	Yes	0.889	Apoplastic
SAMD00023353_4800590	Hypothetical protein	292	No	0.865	Non-apoplastic
SAMD00023353_1401580	Hypothetical protein	230	Yes	0.864	Apoplastic
SAMD00023353_5300760	Hypothetical protein	216	No	0.842	Non-apoplastic
SAMD00023353_2801560	Putative lactoylglutathione lyase	630	No	0.835	Non-apoplastic
SAMD00023353_7700300	Hypothetical protein	207	Yes	0.829	Non-apoplastic
SAMD00023353_1401720	Hypothetical protein	189	No	0.819	Apoplastic
SAMD00023353_9200230	Hypothetical protein	240	Yes	0.815	Non-apoplastic
SAMD00023353_6400250	Hypothetical protein	189	Yes	0.805	Apoplastic
SAMD00023353_0600790	Hypothetical protein	288	No	0.805	Non-apoplastic
SAMD00023353_2100970	Hypothetical protein	150	No	0.804	Non-apoplastic
SAMD00023353_3900800	Cytochrome P450 monooxygenase	360	No	0.802	Non-apoplastic
SAMD00023353_1901790	Hypothetical protein	501	No	0.784	Non-apoplastic
SAMD00023353_13300070	Hypothetical protein	273	No	0.765	Non-apoplastic
SAMD00023353_0104930	Short-chain dehydrogenase reductase	195	No	0.764	Non-apoplastic
SAMD00023353_0103460	Hypothetical protein	336	Yes	0.756	Apoplastic
SAMD00023353_11900020	Glycoside hydrolase	705	Yes	0.753	Apoplastic
SAMD00023353_1700590	Ankyrin repeat domain-containing 52	246	Yes	0.731	Non-apoplastic
SAMD00023353_2400240	Hypothetical protein	585	Yes	0.721	Apoplastic
SAMD00023353_6500130	Hypothetical protein	1340	Yes	0.615	Apoplastic
SAMD00023353_1000090	Hypothetical protein	177	Yes	0.603	Non-apoplastic

^a^Effectors prediction ‘EffectorP 2’ (http://effectorp.csiro.au/). Probability > 60%

Nineteen genes were related to degradation of toxic compounds such as reactive oxygen species (SAMD00023353_5200870), aflatoxins (SAMD00023353_0902760, SAMD00023353_12800020, SAMD00023353_3200110), and antibiotics (SAMD00023353_3600430, SAMD00023353_6600160, SAMD00023353_0702510, SAMD00023353_0100280, SAMD00023353_2201610), among other drugs. *R. necatrix* also over-expressed genes related to transport of toxic compounds, in particular, four (SAMD00023353_2601150, SAMD00023353_2501030, SAMD00023353_3000620 and SAMD00023353_6200040) and two contigs (SAMD00023353_10000080 and SAMD00023353_2200710) showed homology with genes encoding ATP-binding cassette (ABC) transporters and major facilitator superfamily (MFS) transporters, respectively. Expression values of genes homologous to ABC transporters were higher (FC values ranging from 5 to 7) than those observed for MFS transporters (ranging from 2 to 3) (Table 4).

Two genes were selected for being associated with hormone biosynthesis (GA_4_ desaturase family protein SAMD00023353_10100030 and gibberellin 20-oxidase SAMD00023353_1901120) showing FC values of 38.2 and 2.39 respectively and one gene, the argonaute siRNA chaperone complex subunit Arb1 (SAMD00023353_0801000), postulated to play a role in RNA induced transcriptional silencing (Table 4).

The RNAseq analysis also revealed 137 genes only expressed in *R. necatrix* during its growth on avocado roots. From those contigs, 24 were predicted as candidate effector proteins (CEP) by the CSIRO tool EffectorP2 (a machine learning method for fungal effector prediction in secretomes) [19] with a probability above 60% (Table 5). All CEPs, except for SAMD00023353_2100110, SAMD00023353_2801560, SAMD00023353_3900800, SAMD00023353_11900020 and SAMD00023353_1700590, showed no similarity with proteins in the public database. Out of the 24 CEP, 13 were predicted to be secreted by SignalP3 server and ten were determined to have an apoplastic localization by the CSIRO tool ApoplastP (a machine learning method for predicting localization of proteins) [20] (Table 5).

To test any existing relationship within the candidate effectors proteins identified in this study with previously described effectors proteins, the PHI (Pathogen Host Interaction) database was used; i.e., PHI-base is a database of virulence and effector genes that have been experimentally proven via pathogen-host interaction [21]. Blastp was used to match PHI-base with an e-value cutoff of 1E-03 and 30% identity. As result, 3 *R. necatrix* candidate effectors were annotated, SAMD00023353_11900020 encoding a putative glycoside hydrolase, showed the higher percentage of identity with the effector Lysm from *Penicillium expansum* (Identity 44.58%, E-value 9.94 E-53). SAMD00023353_2100110 and SAMD00023353_1700590 showed identity with effectors BEC1040 and Mocapn7 from *Blumeria graminis* (Identity 32.76%, E-value 1.32 E-05) and *Magnaporthe oryzae* (Identity 35.82%, E-value 1.32 E-03), respectively.

## Discussion

Transcriptome analysis of *R. necatrix* strains growing on rich medium, has recently been addressed as an alternative to provide insights into plant pathogenicity mechanisms used by this ascomycete [12, 13]. However, neither of the two studies was carried out using *R. necatrix* directly interacting with a host. This current study fills this gap, obtaining and analyzing the transcriptomes of the virulent CH53 strain during infection of avocado roots and comparing it with that obtained from the fungus cultured in rich medium.

The number of predicted genes (12,104) obtained in this study is congruent with data from previous transcriptomes from *R. necatrix* (10,616 [12];), as well as other plant pathogenic Ascomycota, such as *Fusarium graminearum* (13,332 genes [22];), *Valsa mali* (13,046 genes [11];), or *Magnaporte oryzae* (11,101 genes [23];). When comparing gene expression profiles between *R. necatrix* infecting avocado roots or growing on PDA medium, a number of transcripts were related with major fungal traits involved in the interaction with the host, among others, CWDE [24], production of toxic compounds and detoxification of those produced by the host, or potential effectors.

Phytopathogenic fungi usually produce numerous extracellular enzymes in order to penetrate the host tissue, being cell wall hydrolases and pectinases the most important ones [25]. The high number of CWDE over-expressed during the infection process correlates with previous visualization studies of *R. necatrix* hyphae that directly penetrate through the avocado root cells [9]. In addition, five putative proteases were also identified. Interestingly, gene expression studies carried out on avocado revealed that three protease inhibitors were highly over-expressed in tolerant rootstocks to *R. necatrix* following inoculation with the pathogen but not in susceptible genotypes [10]. This finding suggests that these proteases, up-regulated in *R. necatrix* during the infection process, could play an important role in degrading basal defense proteins on susceptible avocado roots, however, future experiments need to be carried out to confirm this hypothesis.

Several studies support the idea that *R. necatrix* produce toxins that are likely responsible for the symptoms observed in the aerial parts of the plant [26, 27]. Cytochalasin E and rosnecatrone toxins produced by *R. necatrix* [28, 29] are believed to be involved in the onset of disease symptoms in young apple shoots and detached apple leaves [27]. Shimizu et al., [13], identified the cytochalasin biosynthetic gene cluster, containing fourteen genes, within a 36 kb region of the *R. necatrix* strain W97 genome. In the present study, only one gene (putative aflatoxin B1 aldehyde reductase protein) of the putative cytochalasin cluster was highly up-regulated, while it was down-regulated in transcriptomic analyses carried out in the hypovirulent *R. necatrix* strain [13] (Additional file 2). Taking this into consideration, this gene could play an important role in the pathogenicity of *R. necatrix* CH53 on avocado roots, however the role of the cytochalasin E in virulence remains unclear as suggested by other authors [30]. Four more genes related with the production of fungal toxins were up-regulated during the infection process, two of them (putative sterigmatocystin 8-O-methyltransferase and the averantin oxidoreductase) had been previously described to be involved in aflatoxin biosynthesis [31]. Aflatoxins are considered as the most toxic and carcinogenic compounds among the known mycotoxins and 25 clustered genes have been reported to be involved in its biosynthesis [31, 32]. Although the expression of other genes potentially involved in aflatoxin biosynthesis was not observed and no aflatoxin production, even at minimum concentration (< 1 μg/Kg), was detected in wheat grains infected with *R necatrix* (data not shown), future studies should address the detection of this compound on infected roots due to its high toxigenic nature.

As other necrotrophic pathogens, *R. necatrix* seems to have adapted mechanisms to detoxify host metabolites that can interfere with its virulence [33]. Nineteen genes potentially involved in detoxification of antimicrobial compounds were significantly over-expressed. Interestingly, SAMD00023353_12800020 and SAMD00023353_3200110, both repressed in the hypovirulent *R. necatrix* strain [13], showed homology to genes previously described to be involved in detoxification of phytoalexins. The importance of phytoalexin degradation ability in pathogenesis has been proved through transformation experiments [34]. To date, no phytoalexin production has been reported in ‘Dusa’ avocado rootstocks however, mutation experiments of these two genes would be of great interest to reveal their role in degradation of possible fungal toxic compounds produced by avocado roots.

Other contigs were related to transport mechanisms by which endogenous and exogenous toxicants can be secreted. Two major classes of transporter proteins were represented in *R. necatrix* DEGs such as ABC and MFS transporters. Members of both classes can have broad and overlapping substrate specificities for toxic compounds and have been considered as a “first-line fungus defense barrier” [35].

Some necrotrophs are also able to influence host phytohormone levels or employ their own hormone biosynthesis machinery thereby disrupting defense signaling [24, 36–41]. Two genes involved in gibberellin biosynthesis, GA_4_ desaturase family protein and Gibberellin 20-oxidase, were up-regulated during the infection process. Role of GAs in plant-pathogen interactions is not well known [42]; i.e., Studt et al. [43] showed the positive relation between GA production and bakanae disease in rice while Manka [44] found no correlation between GA production and pathogenesis of *Fusarium*.

Throughout the infection process, fungi can actively manipulate host cellular machinery in order to suppress defenses and/or aid disease progression throughout the release of the so-called ‘effector’ proteins [45]. These effectors are usually secreted proteins that act at the host cell surface [46] or are taken up by the plant cell and act internally [47]. In this investigation, a total of 23 genes were predicted to be effectors (with probability above 60%), among which 19 encoded for hypothetical proteins and 10 were predicted as apoplastic effectors, being their place of action the interphase between the hyphae and the host cell. One of the predicted effectors, showed homology to the Lysm1 effector of *Penycilium expansum*. Lysm-containing proteins have been proposed to be involved in binding and sequestering chitin oligosaccharides in order to prevent elicitation of host immune responses [48] and/or to protect fungal hyphae against chitinases secreted by competitors [49]. In this sense, the expression of this effector during *R. necatrix* infection correlates with previous studies in which the overexpression of chitinases on susceptible avocado rootstocks/*R. necatrix* interaction, was reported [10]. Finally, other contig showed homology with the previously described *Blumeria graminis* effector gene BEC1040, which reduces haustoria formation in barley powdery mildew when silenced [50]. These results confirm previous observations by [12], in which BEC1040 homologous effectors in the virulent *R. necatrix* strain KACC40445 were found.

## Conclusion

This study revealed, for the first time, several genes potentially associated with *R. necatrix* pathogenesis on avocado roots. The analysis of the full-length transcriptome of *R. necatrix* during the infection process suggests that the success of this fungus to infect diverse crops might be attributed to a number of produced compounds such as CWDE, toxins, antimicrobial detoxification compounds, transporters, effectors which, singly or in combination, likely interfere with defense or signaling mechanisms found on different plant families [24]. These results are revealing the complexity underlying *R. necatrix* pathogenesis being consistent with the difficulty of WRR management.

Functional characterization of these genes could help to understand how the fungus interferes with the host machinery and the development of white root rot disease. Along this line, a genetic manipulation protocol for transformation of *R. necatrix* has been established, although its efficiency needs to be improved [9]. Nevertheless, the transcriptome analysis of *R. necatrix* during the infection process provides useful information and facilitates further research to a more in -depth understanding of the biology and virulence of this pathogen. In turn, this will make possible to evolve novel strategies for white root rot management in avocado.

## Methods

### Plant material, fungal isolate and inoculation

Clonal 1 year old ‘Dusa™’ plants, described as susceptible to *R. necatrix* [51] and provided by Brokaw nursery (Brokaw España S.L), were potted in 1.5 L plastic pots, previously disinfected with hypochlorite solution (2%) with an sterilized substrate consisting in peat, coconut fibre and perlite mixture (10:10:1) supplemented with 12 g osmocote® and placed into a semi-controlled greenhouse conditions (~ 20 °C temperature and ~ 60% relative humidity). The virulent CH53 fungal strain, isolated at Almuñecar (Granada, Spain) [52], was used in this study and cultured on potato dextrose agar (PDA; Difco Laboratories, Detroit, USA) at 25 °C.

For transcriptome analysis of *R. necatrix* growing on rich medium, the isolate was cultured on PDA covered with a perforated layer of cellophane and incubated 5 days at 25 °C.

For RNA-Seq analysis of *R. necatrix* during infection, plants were removed from the pot and roots were washed with distilled water to remove soil debris. Roots were cut and placed into 15 cm diameter Petri dishes covered with three layers of filter paper soaked with sterilized distilled water. Three perforated cellophane discs, 6 cm diameter, were placed along the roots (Fig. 1). The inoculation was carried out by placing two wheat grains infected with *R. necatrix* onto each cellophane disc. Petri dishes were closed, sealed with parafilm and incubated in dark for 5 days.

### RNA isolation and sequencing

For RNA extractions, cellophane discs covered with grown mycelium, were collected and macerated with liquid nitrogen using a mortar and pestle. One g of frozen powder was collected in a 2 ml Eppendorf and resuspended in 1 ml of denaturation solution (guanidine thiocyanate, 4 M, Na-citrate 25 mM sarcosyl, 0.5%) (Fluka; Switzerland) and saturated phenol pH 4.3 (1:1) plus 7 μl of β-Mercaptoethanol. One hundred μl chloroform were added to the mixture; samples were vortexed and incubated 3 min at room temperature and centrifuged at 12,000 g for 10 min at 4 °C. Afterwards, RNA was extracted using NucleoSpin RNA plant kit (Macherey-Nagel, Germany) following manufacturer’s instructions.

DNAase I (DNase I, Thermo, USA) treatment was carried out twice, during and after the extraction process. RNA quantity and quality were determined based on absorbance ratios at 260 nm/280 nm and 260 nm/230 nm using a NanoDrop® ND-1000 (Nanodrop Technologies, Inc., Montchanin, USA) spectrophotometer. RNA integrity was confirmed by the appearance of ribosomal RNA bands and lack of degradation products after separation on a 2% agarose gel and Red Safe staining.

The integrity of the RNA samples was further verified using the 2100 Bioanalyzer (Agilent Technologies, Inc., Santa Clara, USA) and submitted to the Centre Nacional d’Anàlisi Genòmica (CNAG, Barcelona, Spain) for sequencing. Two μg RNA from each sample were used for RNA library preparation using the TruSeq RNA Sample Preparation Kit (Illumina Inc) according to the protocols recommended by the manufacturer. Each library was paired-end sequenced (2 × 76 bp) by using the TruSeq SBS Kit v3-HS, in a HiSeq2000 platform. More than 40 million reads were generated for each sample. The RNA-Seq reads from six libraries (three biological replicates per condition) were processed to remove adaptor sequences, empty reads, low-quality sequences with a Phred score lower than 20 and short reads (< 25 bp). Resulting reads were stored in FASTQ format. High quality reads were aligned to the *R. necatrix* reference genome [13] for generation of read counts and differential expression analysis. CH53 RNA-seq reads were mapped to the W97 genome and consensus sequences were made of the mapped reads. The overall rate of base changes in the mapped regions between the CH53 and W97 strains was 0.75%. Raw reads from three biological replicates of *R. necatrix* growing on avocado roots and PDA media, are available from the NCBI Gene Expression Omnibus under accession number GSE134243.

A statistical analysis of the expression data of *R. necatrix* growing on avocado roots (RGA) vs Potato Dextrose Agar (RGPDA) media was performed by the Empirical analysis of DGE (EDGE) in CLC Genomics Workbench 10.0.0 (CLC Bio, Aarhus, Denmark). The DEGs were identified using the following conditions: − 2 > fold change > 2 and FDR (*P* < 0.05). A visual representation of DEGs log_10_ FDR *P*-value vs log_2_ Fold change was plotted in R (version 3.6) with a simple scatterplot color coding the different conditions.

### Gene predictions and annotations

*R. necatrix* predicted genes were searched against NCBI Fungi databases to assign associated Gen Ontology (GO) annotations using Blast2Go [18]. GO enrichment analysis (Fisher’s Exact test, [53]) and KEGG pathway analyses were carried out by Blast2go 5.2.4. Default parameters were used with a cut-off FDR of 0.05. GO enrichment analysis (Fisher’s Exact test, [53]) describing the enriched biological processes (BP), molecular functions (MF) and cellular components (CC) of DEGs was performed with B2G according to the following parameters: filter mode as *P*-Value and 0.05 as filter value. Kyoto Encyclopedia of Genes and Genomes (KEGG) annotations [54] of DEGs was performed with B2G.

Genes were clustered using TIGR Multi Experiment Viewer 4.6.1 [55] with Euclidean distances and Average linkage.

SignalP 3.0 server [56] was used to predict the presence and location of signal peptide cleavage sites in amino acid sequences. Localization of proteins to the plant apoplast was predicted by the CSIRO tool ApoplastP [20]. Relationships within the candidate effectors proteins identified in this study with previously described effectors proteins was tested using the PHI (Pathogen Host Interaction) database [21].

### Quantitative real-time PCR

Validation of gene expression levels obtained from the transcriptome analysis was performed using qRT-PCR.

One μg of total RNA was treated with DNase RNase-free (Promega, Madison, USA) following the manufacturer’s instructions. Single-stranded cDNA was synthesized using the iScript cDNA synthesis kit (BIO-RAD, California, USA) following the manufacturer’s instructions. The expression of five *R. necatrix* genes was studied. One endogenous control gene, actin, was used for normalization. Primer sequences for endogenous control gene and the five *R. necatrix* genes are presented in Additional file 3. Primer pairs were chosen to generate fragments between 50 and 150 bp with melting temperature of 60 °C and designed using Primer 3 software [57, 58].

Primer specificity was tested by first performing a conventional PCR and confirmed by the presence of a single melting curve during qRT-PCR. Serial dilutions (1∶10, 1∶20, 1∶50, 1∶200) were made from a pool of cDNA and calibration curves were performed for each gene. The qRT-PCR reaction mixture consisted of cDNA first-strand template, primers (500 nmol final concentration) and SYBR Green Master Mix (SsoAdvanced Universal SYBR Green Supermix, Bio-Rad) in a total volume of 20 μl. The PCR conditions were as follows: 30 s at 95 °C, followed by 40 cycles of 10 s at 95 °C and 15 s at 60 °C. The reactions were performed using an iQ5 real-time PCR detection system (Bio-Rad). Relative quantification of the expression levels for the target was performed using the comparative Ct method [59]. Three biological replicates of RGA or RGPDA vs control samples were performed in triplicate. Statistical significance of the data was determined by a Student’s *t*-test carried out with Sigma Stat version 4.0 software (Systat Software GmbH).

## Supplementary information

**Additional file 1.** Top 20 overexpressed and repressed genes in *R. necatrix* during growth on avocado roots

**Additional file 2.** Genes within the region containing the putative cytochalasin biosynthetic gene cluster in *R. necatrix*

**Additional file 3.** qRT-PCR primer sequences used in this study

## Data Availability

The data from this study are available from the NCBI Gene Expression Omnibus under accession number GSE134243.

## Abbreviations

BP: Biological process
CWDE: Cell wall degrading enzymes
DEG: Differentially expressed genes
FC: Fold change
GO: Gene ontology
KEGG: Kyoto encyclopedia of genes and genomes
MF: Molecular function
PDA: Potato dextrose agar
PHI: Pathogen host interactions
RGA: *Rosellinia necatrix* growing on avocado roots
RGPDA: *Rosellinia necatrix* growing on PDA
WRR: White Root Rot
