# Supplementary material for: Transcriptome analysis of the fungal pathogen Rosellinia necatrix during infection of a susceptible avocado rootstock identifies potential mechanisms of pathogenesis
Source: BMC Genomics. 2019 Dec 26;20:1016. doi: 10.1186/s12864-019-6387-5 (PMC6933693; doi:10.1186/s12864-019-6387-5)
Supplement: Supplementary file 1 — Additional file 1. Top 20 overexpressed and repressed genes in R. necatrix during growth on avocado roots [file 12864_2019_6387_MOESM1_ESM.docx]

**Table S1. Top 20 overexpressed and repressed genes in *R. necatrix* during growth on avocado roots.**

| **Gene ID** | **Description** | ***In vitro* FC^*^** |
| --- | --- | --- |
| ***Up-regulated*** |  |  |
| SAMD00023353_3600480 | Putative geranylgeranyl pyrophosphate synthetase | 1911.00 |
| SAMD00023353_0602170 | Putative carbohydrate-binding module family 1 protein | 1796.59 |
| SAMD00023353_3600440 | Putative cytochrome P450 | 1540.28 |
| SAMD00023353_3600460 | Hypothetical protein | 1438.67 |
| SAMD00023353_10000140 | Putative dj-1 family protein | 1180.90 |
| SAMD00023353_3600470 | Putative cytochrome P450 4V3 | 743.60 |
| SAMD00023353_5900490 | Putative 2`-hydroxyisoflavone reductase | 607.66 |
| SAMD00023353_0503130 | Putative glycoside hydrolase family 61 protein | 511.82 |
| SAMD00023353_1500930 | Putative acid proteinase protein | 411.34 |
| SAMD00023353_3600450 | Putative FAD binding domain-containing protein | 395.89 |
| SAMD00023353_6000590 | Putative lipase 1 precursor | 365.25 |
| SAMD00023353_2301070 | Hypothetical protein | 364.83 |
| SAMD00023353_3600490 | Putative cytochrome p450 | 343.71 |
| SAMD00023353_5500520 | Hypothetical protein | 340.51 |
| SAMD00023353_4400830 | Hypothetical protein | 337.67 |
| SAMD00023353_2301060 | Putative major facilitator superfamily | 300.59 |
| SAMD00023353_3000060 | Putative FAD binding domain-containing protein | 297.12 |
| SAMD00023353_1100630 | Putative cytochrome P450 oxidoreductase | 275.12 |
| SAMD00023353_6500680 | Putative endo beta-glucanase d | 259.37 |
| SAMD00023353_4001240 | Putative glycosyl hydrolase family 43 protein | 226.29 |
| ***Down-regulated*** |  |  |
| SAMD00023353_11200050 | Hypothetical protein | -185.70 |
| SAMD00023353_7000220 | Putative mas3 protein | -137.48 |
| SAMD00023353_7900050 | Hypothetical protein | -120.87 |
| SAMD00023353_3800530 | Hypothetical protein | -82.44 |
| SAMD00023353_3800540 | Hypothetical protein | -80.94 |
| SAMD00023353_2200450 | Putative Amino acid transporter | -65.64 |
| SAMD00023353_2300270 | Hypothetical protein | -45.58 |
| SAMD00023353_2801290 | Putative cell surface protein | -42.34 |
| SAMD00023353_0800590 | Putative carbohydrate esterase family 5 protein | -39.85 |
| SAMD00023353_11500110 | Putative Rodlet peptide | -38.20 |
| SAMD00023353_13900020 | Putative Zinc/iron transporter protein | -37.53 |
| SAMD00023353_0401900 | Hypothetical protein | -36.14 |
| SAMD00023353_11000190 | Putative cell death in tomato 1 | -36.13 |
| SAMD00023353_2800080 | Carbohydrate-binding module family 13 protein | -35.58 |
| SAMD00023353_3000890 | Putative beta-glucosidase M | -34.63 |
| SAMD00023353_0403550 | Putative carbohydrate binding domain-containing protein | -33.75 |
| SAMD00023353_1600920 | Putative pyridoxal-dependent decarboxylase domain protein | -29.14 |
| SAMD00023353_1600930 | Putative pyridoxal-dependent decarboxylase domain protein | -27.11 |
| SAMD00023353_1600940 | Putative pyridoxal-dependent decarboxylase domain protein | -26.33 |
| SAMD00023353_1600210 | Hypothetical protein | -26.19 |

*RNA-Seq fold change, calculated by comparing *R. necatrix* growth on roots vs Potato Dextrose Agar.
