# Supplementary material for: Transcriptome analysis of the fungal pathogen Rosellinia necatrix during infection of a susceptible avocado rootstock identifies potential mechanisms of pathogenesis
Source: BMC Genomics. 2019 Dec 26;20:1016. doi: 10.1186/s12864-019-6387-5 (PMC6933693; doi:10.1186/s12864-019-6387-5)
Supplement: Supplementary file 2 — Additional file 2. Genes within the region containing the putative cytochalasin biosynthetic gene cluster in R. necatrix [file 12864_2019_6387_MOESM2_ESM.docx]

**Table S2. Genes within the region containing the putative cytochalasin biosynthetic gene cluster in *R. necatrix*.**

| **Gene ID** | **Description** | **RGA vs RGPDA FC^a^** | **W97 vs W97 (RnMBV1) FC^b^** | |
| --- | --- | --- | --- | --- |
| SAMD00023353_5500600 | MFS multidrug transporter | -1.44 | | 1.71 |
| SAMD00023353_5500610 | Aflatoxin B1 aldehyde reductase member 2 | **18.65** | | **-2.45** |
| SAMD00023353_5500620 | Putative aldo keto protein | -2.14 | | **-2.00** |
| SAMD00023353_5500630 | Beta-ketoacyl synthase | -1.11 | | **-10.59** |
| SAMD00023353_5500640 | Cytochrome P450 oxidoreductase GliF | -1.29 | | **-2.35** |
| SAMD00023353_5500650 | Major facilitator superfamily transporter | 1.11 | | -1.25 |
| SAMD00023353_5500660 | Alpha/beta hydrolase | -1.03 | | **-7.68** |
| SAMD00023353_5500670 | Cytochalasin biosynthesis protein F | -1.83 | | **-6.87** |
| SAMD00023353_5500680 | Alcohol dehydrogenase superfamily, zinc-type | -1.44 | | **-10.77** |
| SAMD00023353_5500690 | Cytochrome P450 | -1.80 | | **-3.18** |
| SAMD00023353_5500700 | Cytochrome P450 | -1.84 | | **-7.46** |
| SAMD00023353_5500710 | FAD/NAD(P)-binding domain-containing protein | -1.40 | | **-9.65** |
| SAMD00023353_5500720 | C6 finger domain-containing protein | -1.23 | | 1.40 |
| SAMD00023353_5500730 | Hypothetical protein SAMD00023353_5500730 | -3.91 | | -1.86 |

1. ^a^RNA-Seq fold change (FC) values calculated by comparing *R. necatrix* growth on avocado roots (RGA) vs Potato Dextrose Agar medium (RGPDA). ^b^RNA-Seq fold change values from hypovirulent *R. necatrix* strain W97 infected with the megabirnavirus (RnMBV1) [13]. Bold numbers indicate statistically significant results (t-Test, P < 0.05).
