# Supplementary material for: Transcriptome analysis of the fungal pathogen Rosellinia necatrix during infection of a susceptible avocado rootstock identifies potential mechanisms of pathogenesis
Source: BMC Genomics. 2019 Dec 26;20:1016. doi: 10.1186/s12864-019-6387-5 (PMC6933693; doi:10.1186/s12864-019-6387-5)
Supplement: Supplementary file 3 — Additional file 3. qRT-PCR primer sequences used in this study [file 12864_2019_6387_MOESM3_ESM.doc]

**Table S3. qRT-PCR primer sequences used in this study.**

| **Gene ID** | **Sequence** |
| --- | --- |
| Rn-Actin-F | 5´-ATTCAAGCCGTCCTGTCTCTCT-3´ |
| Rn-Actin-R | 5´-CGGAATCCAGCACAATACCA-3´ |
| SAMD00023353_12800020-F | 5´-TATTGCCCTCTCGTCCATTC-3´ |
| SAMD00023353_12800020-R | 5´-TGGGCGTTATCAATCTCCTC-3´ |
| SAMD00023353_2901300-F | 5´-CATCCGCCCTGATAAAAAGA-3´ |
| SAMD00023353_2901300-R | 5´-TCTCCGAGAGCCCCTAAGA-3´ |
| SAMD00023353_2901290-F | 5´-TCGAAGACACTGCCACACTC-3´ |
| SAMD00023353_2901290-R | 5´-GAGTTCCTGAGGCTTGTTGC-3´ |
| SAMD00023353_10000100-F | 5´-TGATAGTTGGCTTCGTGCTG-3´ |
| SAMD00023353_10000100-R | 5´-GGGATATCGCCCACAGTCTA-3´ |
| SAMD00023353_0800710-F | 5´-AAAATCCTGATGCGAAGTGG-3´ |
| SAMD00023353_0800710-R | 5´-TCCGACTGGAAGTTTGATCC-3´ |
